# Microsatellite genotypes of the South African Cape vulture, *Gyps coprotheres*

**DOI:** 10.1038/s41597-019-0221-4

**Published:** 2019-10-11

**Authors:** Courtneë Kleinhans, Sandi Willows-Munro

**Affiliations:** School of Life Sciences, University of KwaZulu-Natal, Pietermaritzburg, South Africa

**Keywords:** Rare variants, Zoology, Genetic markers

## Abstract

Across the globe, vulture species are experiencing major population declines. A key factor for the long-term persistence of these endangered species is the maintenance of genetic diversity patterns within wild populations. The datasets presented in this descriptor includes microsatellite genotypes of 605 Cape vultures (*Gyps coprotheres*) drawn from across the southern African distribution of the species. Microsatellites are useful in quantifying genetic diversity at the population level. Populations of the endangered Cape vulture are currently monitored by conservation agencies and the data presented here can be used as an important baseline for future population genetic monitoring.

## Abstract (summary)

**Table:** 

Measurement(s)	microsatellite
Technology Type(s)	DNA sequencing
Factor Type(s)	geographic location
Sample Characteristic - Organism	Gyps coprotheres
Sample Characteristic - Location	South Africa

Machine-accessible metadata file describing the reported data: 10.6084/m9.figshare.9896372

## Background & Summary

In recent years, Cape vultures (*Gyps coprotheres*) have shown a decline in the overall number of individuals in the wild and their global range is currently undergoing a significant reduction, with most breeding colonies found in South Africa^1–5^. A more rigorous approach is required to successfully stabilize and conserve this endangered vulture^4^. In order to ensure the long-term conservation of vulture populations, management practices should evaluate and maintain the amount and pattern of genetic diversity within current populations^6,7^.

Microsatellites are useful molecular markers used to estimate the amount and pattern of genetic variation at the population-level^8^. These molecular markers show high levels of polymorphism within a species or among populations of the same species^9^. Microsatellites are sensitive to genetic changes such as, changes in effective population sizes and rates of migration among populations^8^ and so can be used to monitor the genetic “health” of populations.

This data descriptor describes a dataset of 605 Cape vultures collected from 24 localities (Supplementary Table 1) genotyped at 13 microsatellite loci. These data were analysed in a recent study that describes the genetic diversity of South African Cape vulture populations^10^ and were used to estimate the regional connectivity of six Cape vulture breeding colonies in South Africa. These data represent an important baseline for future genetic monitoring of wild populations of Cape vulture.

## Methods

### Sampling procedure and sampling localities

A total of 605 Cape vultures from 24 localities, across the South African distribution of the species, were sampled for this study (Supplementary Table 1). This includes 266 samples collected from six breeding colonies. Samples consisted of feather, archival tissue or blood. Feather samples were collected opportunistically from feeding sites, sites of electrocutions, poisoning events and below nests at breeding colonies. Blood samples were collected when vultures were captured and fitted with global positioning system/global system for mobile transmitters^11^. Blood samples were stored on Whatman FTA® Elute cards (Sanford, USA). Archival museum samples (dried skin snips) were sourced from local South African museums (Supplementary Table 1).

### Molecular methods

#### DNA extraction

The NucleoSpin® Tissue kit (Macherey-Nagel, Germany) was used for all DNA extractions. The extraction protocol was modified for feather and archival samples to improve DNA yield. Samples were incubation with proteinase K for 48 hours in a shaking water bath (56 °C), the lysate was incubated in 70 °C B3 buffer for 45 minutes, the final volume of pre-warmed elution buffer (BE) was 80 μl. During the final elution step samples were incubated at 70 °C for 20 minutes followed by centrifuging and then reapplication of the solution onto the membrane. The samples were incubated again at 70 °C for an additional five minutes followed by the final centrifuging step.

#### Microsatellite amplification

Thirteen microsatellite loci were selected from previous studies^12,13^ (Table 1). Each primer was fluorescently labeled, using three-fluorophore analogues, according to their expected allelic size and sequence motif (Table 1). Six multiplex reactions where designed according to microsatellite loci amplification, fluroscent dye and optimal annealing temperature (Table 1). All samples were amplified in six multiplex reactions using the KAPA2G^TM^ Fast Multiplex PCR kit (KAPA Biosystems) following the manufacture’s protocol. All amplified products were analyzed using a 3130xL Genetic Analyzer housed at the Central Analytical Facility at Stellenbosch University, South Africa. The software GeneMarker v2.4.0 (Soft Genetics) was used for genotype scoring^14,15^.

**Table 1:** Details of microsatellite loci used to amply 605 Cape vultures *Gyps coprotheres*.

Locus	Sequence	Motif	Label	Allele Size range (bp)	Multiplex Reaction
BV2	F: CAGCATGTTATTTTGGCTGC	(CA)_11_	HEX	110–136	Multiplex 6
	R: TTGCTAAACCGGTTAGAAGTTG				
BV5	F: GTTCTGAGGGTAGAGGGACTG	(CA)_17_	Tet	166–182	Multiplex 1
	R: GCTGAGCAGCTTCAGAAAGTC				
BV6	F: AATCTGCATCCCAGTTCTGC	(CA)_11_	HEX	100–150	Multiplex 4
	R: CCGGAGACTCTCAGAACTTAAC				
BV9	F: ATCTAGGGACATCGAGGAGC	(TA)_6_(CA)_11_	HEX	196–384	Multiplex 6
	R: ACAGGGATGCAGGTAAGCC				
BV11	F: TGTTTGCAAGCTGGAGACC	(CA)_22_	HEX	146–186	Multiplex 3
	R: AAAAGCCTTGGGGTAAGCAC				
BV12	F: TCAGGTTTTGACGACCTTCC	(CA)_15_	6-Fam	240–290	Multiplex 2
	R: GTGGTAACGGAGGAACAAGC				
BV13	F: AAAACAGAGTTTTCACATTTTCATAAG	(CA)_16_	6-Fam	163–187	Multiplex 3
	R: TTCAGGAAACAGAAGCATGAAC				
BV14	F: GGCAGTGTGGAGCCTACATC	(CA)_16_	6-Fam	148–186	Multiplex 4
	R: CTCCAGGGTCCTTGTTTGC				
BV20	F: GAACAGCACTGAACGTGAGC	(CA)_13_	HEX	133–195	Multiplex 1
	R: GTTTCTCCTGACAGTGAAATAACTC				
Gf3H3	F: GTAGAATAATTTGCTCCTGG	(CT)_12_	6-Fam	123–197	Multiplex 2
	R: GTGAAGGCACCTCATAGACA				
Gf8G	F: TGAGCAGGTGAGTCCAGAAG	(CT)_8_C (TC)_2_	6-Fam	226–292	Multiplex 4
	R: GCTCTCCTGTCATCTTGCAT				
Gf9C	F: GGTGGACATTACATACACTG	(TC)_10_ + (CT)_9_ C (CA)_5_T (AC)_4_	HEX	217–315	Multiplex 3
	R: CAAGGAATCTGGACTACTAA				
Gf11A4	F: GATCCCTTCCAACCGAAAAT	(CTCTT)_17_	HEX	110–160	Multiplex 2
	R: TGGTGACCAACGGAAGTGTG				

## Data Records

The datasets are available on Zenodo and include the raw fragment analysis data for the 605 *Gyps coprotheres* genotyped using 13 microsatellite loci^14^ as well as the genotype scores for the 605 *Gyps coprotheres* individuals^15^. All associated metadata (tissue type, sampling locality, and date of collection) is available in Supplementary Table 1. Multilocus microsatellite alleles are scored according to size in base pairs and missing data is encoded as “0”. Percentage of missing data included in the final dataset varied across loci (Supplementary Table 2) but was minimal (mean = 11%).

## Technical Validation

To ensure genotype data quality, all archival samples were re-amplified, and each locus was genotyped multiple times (up to five times) and compared for consistency. In addition, 20% of all feather, muscle and blood samples were re-amplified multiple times (up to five times) to verify the reliability of the data. Negative controls were including in each genetic analyzer run to check for contamination of reagents. When consistent genotypes were not generated the genotype scores was inputted as missing data.

We used identity analysis in the software Cervus v3.0.7^16^ to ensure that duplicated genotypes were not included in the final data. This is particularly important in this study as genotypes were amplified from discarded feathers collected at colonies. Null alleles can be a problem in studies that use primers not designed for the study species and this can bias population structure analysis^17^. Uncorrected global F_ST_ were compared to F_ST_ values corrected using the excluding null alleles (ENA) method^18^ using a paired t-test. The paired t-tests were not significant (p-value > 0.05) suggesting that these data are not affected by null alleles.

## Supplementary information

Supplementary Table 2.

Supplementary Table 1.
